# Non-scarring alopecia in systemic lupus erythematosus patients at the Lagos State University Teaching Hospital: a cross-sectional study of prevalence, pattern, trichoscopy features and histopathological analysis

**DOI:** 10.11604/pamj.2024.47.9.33647

**Published:** 2024-01-09

**Authors:** Anaba Ehiaghe Lonia, Olaosebikan Hakeem, Cole-Adeife Olufolakemi, Olayemi Olubunmi Dawodu, Olufemi Adelowo

**Affiliations:** 1 Department of Medicine, Lagos State University Teaching Hospital, Lagos, Nigeria,; 2 Department of Anatomic and Molecular Pathology, College of Medicine, University of Lagos, Lagos, Nigeria

**Keywords:** Trichoscopy, hair loss, histopathology, systemic lupus erythematosus

## Abstract

**Introduction:**

trichoscopic and histopathological evaluation of non-scarring systemic lupus erythematosus (SLE) alopecia is uncommon. We aimed to document the prevalence, pattern of hair loss, trichoscopic and histopathologic differences between systemic lupus erythematosus patients with and without hair loss.

**Methods:**

this was a cross-sectional comparative study of 75 systemic lupus erythematosus patients, 36 with hair loss from February to December 2020. Trichoscopic evaluation was conducted on all 75 patients. Twenty-three patients (12 with hair loss and 11 without) had scalp biopsies with mucin deposit evaluation. Disease activity was documented using the SLE disease activity index. Data was analyzed using SPSS 22.

**Results:**

the mean age of the patients was 33.7 ± 12.4 years. Non-scarring alopecia was observed in 48%. The pattern of hair loss was <4 patches in 44.4%, mild diffuse in 25%, and severe diffuse in 30.6%. Disease activity was mild in 38.9%. Hair shaft changes included thin hair (97.2%), decreased number of hairs per follicular unit (97.2%), hypopigmented hair (85.7%), and follicular red dots (27.8%). Significant differences between the two groups were; a reduction in size and number of sebaceous glands on histopathology, hair shaft, and scalp pigmentary changes in the hair loss group.

**Conclusion:**

the prevalence of non-scaring alopecia is high in SLE patients with patchy type as the commonest pattern. Trichoscopic and histopathologic differences exist in SLE patients with and without hair loss and the normal-appearing scalp in SLE patients is involved in the inflammatory process. Hair shaft thinning, hypopigmentation, and scalp pigmentary changes occur in SLE.

## Introduction

Systemic lupus erythematosus is a multi-systemic autoimmune inflammatory disease that affects every organ in the body including the skin and hair [1-4]. The prevalence of SLE in Africa is reported to be 0.3/100,000 per person per year and SLE accounts for 5.4 to 91% of rheumatological diseases in Nigeria [2,5-7]. Systemic lupus erythematosus affects more females than males especially females in their child bearing age [2,6,8,9]. The few studies on hair disorders in SLE showed that, the hair is affected in 46.8 to 85.2% of patients and that, hair involvement is associated with increased disease severity [4,6,8,9].

The predominant hair disorders seen in SLE are non-scarring alopecia, hair shaft, and scalp abnormality without apparent hair loss [3,4,6,8-10]. Non-scarring alopecia is found in over 80% of SLE patients [6,9] and this is documented to be mild diffuse in 43.8 to 65%, severe diffuse in 7.3 to 15.6%, patchy in 11 to 28%, and lupus hair in 4.5% [4,8,9]. The pattern of non-scarring alopecia can be used in the assessment of SLE flares as severe diffuse non-scarring alopecia indicates an active disease [4].

Tricohoscopy features of SLE reported in various studies include hair thinning, incomplete alopecia, hair shaft hypopigmentation, telangiectasias, peripilar sign, perifollicular red dots, white dots, and honeycomb pigment pattern [3,4,8-11]. Other follicular dots include black dots, yellow dots, blue-grey speckled and brown scattered pigmentation [3,4]. Histopathology evaluation of SLE is lichenoid with epidermal atrophy, basal vacuolization, perifollicular and perivascular lymphocytic infiltrates, pigment incontinence, increased catagen/anagen hair follicle numbers and a reduced number of sebaceous glands [9,11].

Non-scarring alopecia is one of the parameters used in the assessment of SLE disease activity and this is included in the systemic lupus erythematosus disease activity index instrument (SLEDAI) [4,12,13]. The SLEDAI is used routinely in the assessment of SLE patients and is made up of 24 parameters covering physical and laboratory parameters with a total score of 105 [13]. A disease flare is reported when the SLEDAI score is ≥6 [14,15].

Studies of the pattern of non-scarring alopecia in SLE patients, the trichoscopy and histopathology features are uncommon in our environment. We do not know if SLE already affects the scalp of individuals without apparent hair loss. It is our hypothesis that, there are both trichoscopic and histopathological differences between SLE patients with and without hair loss and that, SLE patients without hair loss may have histopathologic evidence of hair follicle inflammation.

**Objectives:** the objectives of this study were to document the prevalence, pattern, trichoscopy and histopathologic features of non-scarring alopecia in SLE patients and to correlate alopecia with the severity of SLE disease using the SLEDAI. Additionally, we sought to compare trichoscopy and histopathology features in SLE patients with and without non-scarring alopecia and, to compare trichoscopy and histopathology features in alopecic patches with non-alopecic areas of the scalp in SLE patients and to correlate findings with SLE disease activity (SLEDAI). It is our hypothesis that, there will be differences in trichoscopy and histopathology features between SLE patients with and without hair loss. Our second hypothesis was that in SLE patients who have hair loss, the areas of the scalp without hair loss may have histopathologic evidence of inflammation as SLE is a systemic disease.

**Research question:** 1) What are the clinical patterns of hair loss in SLE patients? 2) Are there any differences in trichoscopy and histopathology features between SLE patients with and without hair loss? 3) Is there any histopathologic evidence of inflammation in the normal-appearing scalp of SLE hair loss patients?

## Methods

**Study design:** this was a cross-sectional descriptive study of 75 adult SLE patients.

**Setting:** the study was conducted in 2020 at the Rheumatology Clinic of the Lagos State University Teaching Hospital following ethical approval by the health research and ethics committee of the hospital (LREC/06/10/1299). The study was conducted over a six-month period (February, July to December 2020) There was a temporary break in the study due to the COVID-19 pandemic.

**Participants:** seventy-five (75) consenting SLE patients who met the American Academy of Rheumatology criteria for SLE diagnosis [16] and attended the clinic during the study period were consecutively recruited into the study. Excluded from the study were SLE patients who had scarring alopecia (discoid lupus erythematosus), SLE patients who had other scalp conditions that could cause hair loss (seborrheic dermatitis, psoriasis, lichen planopilaris). Also excluded were patients who had other autoimmune or medical diseases that can affect the hair and scalp (hyper/hypothyroidism, pernicious anaemia) and patients who had a change in medications within 3 months of the study as the drugs can cause telogen effluvium (TE).

**Variables:** a study questionnaire (socio demographic and clinical data; (Annex 1) designed for this study was administered to each patient. All the patients were clinically examined for alopecia, the pattern of alopecia, the colour, and the thickness of the hair. In this study, alopecia was categorized as [11]: patchy if less than 4 areas of separated alopecia; mild diffuse if there is a widening of the central part of the scalp; severe diffuse if the affected area is more than 50% of the scalp. Hair was regarded as thin/lupus if short, coarse, fragile, and along the anterior hair line [4].

Hair shaft changes were defined as [4]: decreased in number if only a single hair is observed in each follicular opening in more than 20% of the follicles/field of vision; decreased in diameter if differences in hair diameter is observed in more than 20% of the hair follicles/field of vision; hypopigmented if hypopigmented hair shafts are observed in at least 20% of observed hair. Trichoscopy examination was conducted on all the patients irrespective of hair loss using the DermLite DL4 dermotoscope (3Gen Inc., San Juan Capistrano, CA, USA) with an x10 magnification. Five areas of the scalp in each patient were examined in the non-polarized mode; frontal, left and right temporal, vertex, and occipital by two board-certified dermatologists and documented as follows: scalp pigmentary changes; black dots, yellow dots, honey comb pigment, brown scattered pigmentation, brown peripilar sign, follicular red dots and blue-grey pigmentation; arborizing blood vessels (telangiectasia); as thin if the blood vessels are thinner than the diameter of terminal hair shafts, prominent if same size as terminal hairs and forming visible vascular networks regularly between follicular units, thick arborizing if blood vessels are larger than the diameter of terminal hair shafts.

**Figure 1:**
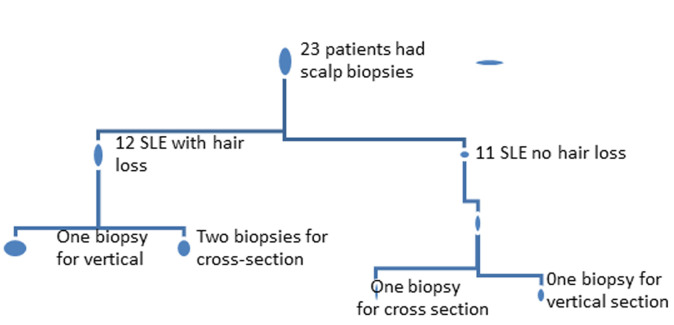
flow chart of scalp biopsy for histopathology

Each patient was examined in a sitting position with good natural light. The dermoscope was lightly applied to the scalp and trichoscopy features were documented. Patients who had alopecia had comparative areas of the scalp examined to determine any differences in trichoscopy features between alopecic and non-alopecic scalp areas. So, if a patient had an alopecic patch on the frontal area; this patch and the adjacent non-alopecic area were examined. Histopathology evaluation was done on 23 (12 with alopecia and 11 without alopecia) consenting patients. Scalp biopsy was performed using a disposable 4 mm punch biopsy needle. In each patient that had alopecia, three scalp biopsy specimens were obtained; 2 from the area of hair loss and one from a non-alopecic area for a comparative cross-sectional study (Figure 1).

The biopsied area for hair loss was occipital in patients with diffuse alopecia and on the most severely involved areas in patients with localized alopecia. In patients without alopecia, 2 biopsies were taken from the occipital region (for vertical and cross-sectional studies) (Figure 1). Cross-sectioning was done according to the description by Headington [17]. Both the vertical and cross-sectional specimens were stained with hematoxylin and eosin. The vertical sections were evaluated for epidermal atrophy, interface changes, type of inflammatory infiltrates, morphology of hair follicles, and size of sebaceous glands. The cross-sectioned specimens were evaluated for follicular counts, inflammatory infiltrates, and size of sebaceous glands. Additionally, an Alcian blue stain (pH 2.5) for mucin evaluation was done. The degree of mucin deposition was categorized as mild (sparse deposits), moderate (focal collection), or severe (significant deposits leading to separation of the collagen bundle) [9,18].

The activity of SLE was documented using the SLE disease activity index (SLEDAI, Annex 2) [13]. The SLEDAI is made up of 24 parameters with weighted scores ranging from 2 to 8 covering physical and laboratory parameters with a total score of 105 [13]. The degree of activity on the SLEDAI was graded as not active, mild, moderate, and severe respectively with scores of 0, 1-5, 6-12, and ≥13 respectively, and patients with scores ≥6 are regarded as having a flare [13-15].

**Bias:** the study was conducted using a questionnaire with defined and standardized features (Annex 2) from the literature with references [13-15]. This was to eliminate any subjectivity/bias. Also, the SLEDAI used in SLE activity assessment is a well-validated questionnaire [13-15].

**Study size:** based on a 48% prevalence of hair loss in their study on the cutaneous manifestation of SLE by Ayanlowo *et al*. [6] a sample size of 124 was calculated for this study. However, only 75 SLE patients were available for study in the clinic.

**Quantitative variables:** data was entered and analyzed using the IBM statistics version 22. A test of normality for numerical variables was used to determine variables that were normally distributed.

**Statistical methods:** the normality of the continuous variables was tested using the Kolmogorov-Smirnov test. The continuous variables such as duration of SLE, duration of treatment, and follicular characteristics that were skewed were presented as median and interquartile range. Age and SLEDAI scores were presented as mean and standard deviation because they were normally distributed. The Student ‘t’ test was used to compare the means of the two groups (SLE patients with and without hair loss), while the median of the two groups was compared using the Mann-Whitney U test and the Kruskal Wallis test respectively. To compare categorical variables between the groups, the Chi-squared test and Fischer´s exact test as the case may be. P value < 0.05 was considered significant for all statistical tests.

## Results

**Participants:** seventy-eight patients who met the diagnostic criteria for SLE were attended to during the study period and 3 of them were excluded from the study for having other hair disorders, leaving 75 patients for the study. Twenty-three patients underwent scalp biopsies; 12 who had SLE hair loss and 11 who did not have hair loss for comparison. Only 23 patients consented to having scalp biopsies. A total of 58 scalp biopsies were obtained.

**Descriptive data:** the mean age of the patients was 33.7 ± 12.4 years, age range of 12-75 years, and 98.7% were females. Systemic LE was more prevalent in those aged 20-39 years and the mean duration of SLE was 3.4 ± 3.3 years. Cutaneous involvement was clinically observed in 24% (18/75) and this was acute, subacute, and lichenoid in 66.7%, 22.2%, and 11.1% respectively. Other organs involvement were musculoskeletal in 64%, renal in 37.3%, and central nervous system in 14.7%.

**Outcome events:** thirty-six patients had hair loss. Hair shaft changes were observed on trichoscopy in 35 of these hair loss patients. Typical lichenoid inflammation was seen on histopathology.

### Main results

**Patients with non-scarring alopecia:** non-scarring alopecia was clinically observed in 48% (36/75) of the patients. The mean age of those who had hair loss was 34.6 ± 14.3 years and 31.1 ± 9.9 years in those who did not have hair loss. Median (IQR) duration of hair loss was 1 (0.1, 17) years. Hair loss was still on-going in 54.6% (17/36) patients and a family history of hair loss was reported in 16.7% (6/36). The pattern of hair loss was <4 patches in 44.4% (16/36), mild diffuse in 25% (9/36), and severe diffuse in 30.6% (11/36). Disease activity (SLEDAI) was mild in 38.9% (Table 1).

**Table 1:** socio-demographic and trichoscopy features of SLE patients with and without hair loss

Variable	Hair loss n (%) = 36	No hair loss n (%) = 39	P
**Age group (years)**			
<20	3 (8.3)	5 (12.8)	
20 - 29	11 (30.6)	12 (30.8)	
30 - 39	10 (27.8)	16 (41.0)	
40 - 49	7 (19.4)	4 (10.3)	
≥50	5 (13.9)	2 (5.1)	
Age (in years), mean (sd)	34.6 (14.3)	31.1 (9.9)	0.054
Age range	12-75	14-54	
Gender, M:F	0:36	1:38	1.000*
Duration of SLE: years, median (range)	3 (0.2, 16)	2 (0.0, 16)	0.841
Family history of hair loss	6 (16.7)	1 (2.6)	0.050*
Still losing hair	17 (54.6)		
Growth following treatment	17 (54.6)		
On treatment for SLE, n (%)	32 (88.9	29 (74.4)	0.107
Treatment duration (years) median (IQR)	2.0 (0.4, 5.0)	2 (1, 4.5)	0.896
**Trichoscopy findings**			
Preserved honeycomb pigmentation, n (%)	30 (83.3)	38 (97.8)	0.050*
**Hair shaft changes**			
Short re-growing hair, n (%)	11 (30.5)	1 (2.6)	0.001*
Hair shaft thinning, n (%)	35 (97.2)	1 (2.6)	<0.001*
Hair shaft hypopigmentation, n (%)	30 (85.7)	1 (2.6)	<0.001*
**Follicular opening/dots**			
Black dots, n (%)	6 (16.7)	1 (2.6)	0.050*
White dots, n (%)	35 (97.2)	38 (97.4)	1.000*
Follicular red dots, n (%)	10 (27.8)	0 (0.0)	0.001*
Blue grey pigmentation, n (%)	4 (11.1)	0 (0.0)	0.048*
Brown scattered pigmentation	16 (44.4)	2 (5.1)	<0.001*
Arborizing/ interconnecting vessels, n (%)	10 (100.0)	0 (0.0)	<0.001*
Thick vessels, n (%)	4 (11.1)	0 (0.0)	0.048*
**SLEDAI scores**			
SLEDAI score mean (SD), range	8.8 (7.6)	8.3 (7.9)	0.817
SLEDAI score ≥6, n (%)	15 (41.7)	21 (53.8)	0.292
CNS domain, n (%)	6 (16.7)	8 (20.5)	0.669
MSS domain, n (%)	8 (22.2)	17 (43.6)	0.050
Renal domain, n (%)	14 (38.9)	14 (35.9)	0.789
Cutaneous domain, n (%)	33 (91.7)	4 (10.3)	<0.001*
Serositis domain, n (%)	0 (0.0)	3 (7.7)	0.241*
Immunologic domain, n (%)	6 (16.7)	6 (15.4)	1.000*
Haematologic domain, n (%)	1 (2.8)	1 (2.6)	1.000*
Vasculitis domain, n (%)	1 (2.8)	0 (0.0)	0.480
Fever domain, n (%)	3 (8.3)	2 (5.1)	0.666*

* : Fischer’s exact p-value; SLE: systemic lupus erythematosus; SLEDAI: systemic lupus erythematosus disease activity index instrument; CNS: central nervous system; MSS: musculoskeletal system;

**Trichoscopy features:** hair shaft changes were thin hair in 97.2% (35/36), decreased follicular unit in 97.2% (35/36), hypopigmented hair in 85.7 (30/36), short re-growing hair in 30.5% (11/36) and lupus hair in 88.9% (32/36) (Table 1). Follicular red dots were observed in 27.8% (10/36), thick arborizing vessels in 11.1% (4/36), and peripilar brown pigments.

**Comparison between SLE patients with and without non-scarring hair loss:** there were no significant differences in socio-demographic factors between the two groups. Significant differences were observed in trichoscopy features; hair shaft and scalp pigmentary changes. Only those with hair loss had follicular red dots. A summary of comparative findings is detailed in Table 1.

**Histopathology and trichoscopy in those who had a biopsy (alopecic scalp):** histopathology findings in the alopecic scalp of the hair loss group included; epidermal atrophy, perivascular lymphocytic infiltrates, severe mucin deposition (41.7%), reduction in size and number of sebaceous glands (58.3%), increased telogen percentage, reduced anagen counts, decreased follicular counts and preserved follicular units. With the exception of sebaceous glands, the histopathology differences between the two groups did not attain statistical significance. There were significant trichoscopy differences in the two groups; hair shaft pigmentation, brown pigments, and follicular red dots (Table 2).

**Table 2:** trichoscopy and histopathology findings in those with and without hair loss who had scalp biopsy

Variable	Hair loss group n=12 (%)	No hair loss group n=11 (%)	P
**Trichoscopy findings**			
**Hair shaft changes**			
Hair shaft thining, n (%)	2 (16.7)	0 (0.0)	0.478*
Short regrowing hair, n (%)	3 (25.0)	0 (0.0)	0.217*
Hair shaft hypopigmentation, n (%)	9 (75.0)	1 (9.1)	0.003*
**Follicular opening/dots**			
Black dots, n (%)	1 (8.3)	0 (0.0)	1.000*
White dots, n (%)	12 (100.0)	11 (100.0)	undefined
Follicular red dots, n (%)	5 (41.7)	0 (0.0)	0.037*
Blue grey pigmentation, n (%)	2 (16.7)	0 (0.0)	0.478*
Brown scattered pigmentation	7 (58.3)	0 (0.0)	0.005*
Arborizing/ interconnecting vessels,	4 (33.4)	0 (0.0)	0.090*
**Histology**			
Epidermal atrophy n (%)	6 (50.0)	2 (18.2)	0.193*
**Interface change**			
Along dermoepidermal junction, n (%)	1 (8.3)	0 (0.0)	0.193*
Along follicular epithelium, n (%)	1 (8.3)	0 (0.0)	1.000*
Lymphocytic infiltration, n (%)	4 (33.3)	1 (9.1)	0.317*
Superficial perivascuylar, n (%)	3 (25.0)	1 (9.1)	0.590*
Perifollicular, n (%)	1 (8.3)	0 (0.0)	1.000*
Presence of plasma cells, n (%)	1 (8.3)	0 (0.0)	1.000*
Reduction in size/ number of sebaceous gland	7 (58.3)	1 (9.1)	0.022*
**Mucin deposition**			
Mild	2 (16.7)	4 (36.4)	
Moderate	4 (33.3)	5 (45.5)	0.356
Severe	5 (41.7)	2 (18.2)	
**Folicular characteristics**			
Tellogen counts, median (range)	3 (1 - 6)	2 (0 - 6)	0.556
Number of hairs, median (range)	16 (4 - 30)	25 (5 - 34)	0.066
Follicular units, median (range)	8 (3, 13)	10 (2 - 12)	0.150
Anagen follicle, median (range)	15 (3 - 25)	21 (2 - 30)	0.250
Telogen percentage, median (range)	15 (4 - 57)	8 (0 - 26)	0.139
Terminal: vellus ratio, median (range)	9 (2 - 23)	7 (4 - 20)	0.719

* : Fischer’s exact p-value

**Comparison of normal-appearing scalp areas in the SLE hair loss group to the normal scalp in those without hair loss:** histopathology of the normal-appearing scalp in the non-scarring hair loss group was; epidermal atrophy in 50%, lymphocytic infiltrates in 33.3% and this was superficial perivascular in 25%, and reduced number and size of sebaceous glands in 58.3%. There was increased telogen percentage, reduced anagen hair follicles, preserved follicular units, and the presence of mucin. The only significant histopathologic difference between the two groups was in the number and size of sebaceous glands. There were significant differences in trichoscopy features; hair shaft pigmentation, and presence of white dots (Table 3).

**Table 3:** trichoscopy and histopathology features of normal appearing scalp in SLE patients with and without hair loss who had scalp biopsy

Variable	Normal appearing scalp in SLE hair loss group n=12 (%)	No hair loss group n=11 (%)	p
**Trichoscopy findings**			
**Hair shaft changes**			
Short regrowing hair, n (%)	3 (25.0)	0 (0.0)	0.217*
Hair shaft hypopigmentation, n (%)	9 (75.0)	1 (9.1)	0.003*
**Follicular opening/dots**			
White dots, n (%)	2 (16.7)	11 (100.0)	<0.001
Brown scattered pigmentation, n (%)	1 (8.3)	0 (0.0)	1.000*
Arborizing/ interconnecting vessels, n (%)	1 (8.3)	0 (0.0)	1.000*
**Histology**			
Epidermal atrophy, n (%)	6 (50.0)	2 (18.2)	0.193*
Interface change			
Along dermoepidermal junction, n (%)	1 (8.3)	0 (0.0)	1.000*
Along follicular epithelium, n (%)	1 (8.3)	0 (0.0)	1.000*
Infiltrates			
Lymphocytic infiltrates, n (%)	4 (33.3)	0 (0.0)	1.000*
Superficial perivascular, n (%)	3 (25.0)	0 (0.0)	1.000*
Perifollicular, n (%)	1 (8.3)	1 (9.1)	0.317*
Presence of plasma cells, n (%)	1 (8.3)	1 (9.1)	0.590*
Sebaceous glands			
Reduction in size/ number of sebaceous gland	7 (58.3)	1 (9.1)	0.027*
Mucin deposition			
Mild	5 (41.7)	4 (36.4)	
Moderate	5 (41.7)	5 (45.5)	0.967
Severe	2 (16.7)	2 (18.2)	
Follicular characteristics			
Telogen counts, median (range)	2.5 (0 - 9)	2 (0 - 6)	0.478
Number of hairs, median (range)	18 (7 - 35)	25 (5 - 34)	0.308
Follicular units, median (range)	8.5 (5 - 13)	10 (2 - 12)	0.596
Anagen follicle, median (range)	14 (3 - 30)	21 (2 - 30)	0.423
Telogen percentage, median (range)	17 (0 - 62)	8 (0 - 26)	0.085
Terminal: Vellus ratio, median (range)	7 (4 - 17)	7 (4 - 20)	0.803

* : Fischer’s exact p-value

There were no significant trichoscopy or histopathology differences between the hair loss types (Table 4). There was significant disease activity based on the SLEDAI scores in those who had hair loss (Table 5). There was no difference in activity between the different hair loss types.

**Table 4:** comparison of features between the different types of non-scarring hair loss

Variable	Patchy (n-16)	Mild diffuse (n = 9)	Severe diffuse (n = 11)	p
**Sociodemographic**				
Age (years) mean (sd)	34.4 (12,2)	34.4 (15.8)	41.4 (15.9)	0.409
Duration of SLE, years, median (range)	3 (0.3, 8)	5 (1.0, 16)	0.7 (0.3-8.0)	0.025
Duration of hair loss years, median (range)	1 (0.3,	2 (0.5 - 17)	0.7 (0.1-5.0)	0.418
Duration of SLE treatment, median (range)	2.5 (0.1, 8)	3.5 (0.5 -15)	3.5 (0.3-12)	0.043
**Trichoscopic findings**				
**Hair shaft changes**				
Short re-growing hair, n (%)	2 (12.5)	(44.4)	5 (45.4)	0.108
Hair shaft thining, n (%)	0 (0.0)	2 (22.2)	3 (27.3)	0.310
Hair shaft hypopigmentation, n (%)	1 (6.3)	2 (22.2)	2 (18.2)	0.479
**Follicular opening/dots**				
Black dots, n (%)	3 (18.8)	1 (11.1)	2 (18.2)	0.874
White dots, n (%)	16 (100.0)	9 (100.0)	10 (90.9)	Invalid
Follicular red dots, n (%)	4 (25.0)	3 (33.3)	3 (27.3)	0.904
Blue grey pigmentation, n (%)	1 (6.3)	0 (0.0)	3 (27.3)	0.110
Brown scattered pigmentation, n (%)	3 (18.8)	4 (44.4)	9 (81.8)	0.005
Arborizing/ interconnecting vessels, n (%)	2 (12.5)	3 (33.3)	5 (45.4)	0.156
Thick vessels, n (%)	2 (12.5)	0 (0.0)	2 (18.2)	0.881
**Histology**				
Epidermal atrophy, n (%)	1 (33.3)	2 (66.7)	3 (50.0)	0.717
Interface change, n (%)	0 (0.0)	0 (0.0)	2 (33.3)	0.301
Along dermoepidermal junction, n (%)	0 (0.0)	0 (0.0)	1 (16.7)	0.580
Along follicular epithelium, n	0 (0.0)	0 (0.0)	1 (16.7)	0.580
Lymphocytic infiltration, n (%)	0 (0.0)	1 (33.3)	3 (50.0)	0.325
Superficial perivascuylar, n (%)	0 (0.0)	1 (33.3)	2 (33.3)	0.513
Perifollicular,n (%)	0 (0.0)	0 (0.0)	1 (16.7)	0.580
Presence of plasma cells, n (%)	1 (33.3)	0 (0.0)	0 (0.0)	0.195
Reduction in size/ number of sebaceous gland	1 (33.3)	3 (100.0)	3 (50.0)	0.214
**Mucin deposition**				
Mild	0 (0.0)	0 (0.0)	2 (33.3)	
Moderate	0 (0.0)	2 (66.7)	2 (33.3)	0.134
Severe	3 (100.0)	1 (33.3)	2 (33.3)	
**Follicular features**				
Telogen count	5 (1 - 6)	1 (1- 3)	5 (1 - 5)	0.309
Number of hairs, median (range)	14 (11 - 24)	10 (4 - 13)	22 (16 - 30)	0.047
Follicular units, median (range)	7 (5 - 11)	4 (3 - 8)	10 (7 - 13)	0.150
Anagen follicle, median (range)	10 (6 - 19)	9 (3 - 10)	21.5 (18 - 25)	0.050
Telogen percentage, median (range)	21 (9 - 57)	18 (8 - 25)	9 (4 - 16)	0.201
Terminal: Vellus ratio, median (range)	4 (2 - 6)	9 (4 - 13)	10 (5 - 23)	0.100

**Table 5:** association of SLEDAI scores and hair loss

Variable	Hair loss n =36 (%)	No hair loss n = 39 (%)	p
**SLEDAI**			
Range	0 - 25	0 - 28	
Mean (SD)	8.8 (7.6)	8.3 (7.9)	0.817
Median (IQR)	6 (2, 15)	8 (0, 14)	0.705
**SLEDAI grade**			
No activity	1 (2.8)	10 (25.6)	
Mild	14 (38.9)	6 (15.4)	0.023
Moderate	11 (30.6)	11 (28.2)	
High	4 (11.1)	7 (17.9)	
Very high	6 (16.7)	5 (12.8)	
**SLE flare**			
SLE scores ≤ 6	21 (58.3)	18 (46.2)	0.292
SLE score > 6	15 (41.7)	21 (53.8)	
**Pattern of hair loss**			
Patchy	5 (2, 17)	0.644	
Mild diffuse	8 (3, 15)		
Severe diffuse	6 (4, 16)		

SLEDAI: systemic lupus erythematosus disease activity index instrument; SLE: systemic lupus erythematosus

## Discussion

This study demonstrates predominance of SLE non-scarring alopecia in young females, its trichoscopy and histopathology features. Also, demonstrated are; differences in trichoscopy and histopathology features between SLE patients with and without alopecia, and pathological involvement of the normal appearing scalp of SLE hair loss patients.

Systemic lupus erythematosus was more prevalent in individuals aged 20-39 years of age and almost all the patients studied were female. This age and gender result is in keeping with SLE being a disease with a penchant for females in their child bearing age [1,6,8,9]. Except for a family history of hair loss, there were no significant socio-demographic differences between patients with and without alopecia. In a similar study by Chanprapaph *et al*. no differences in socio-demographic features were recorded between SLE patients with and without alopecia although in their study, a family history of hair loss was not reported [9].

Non-scarring alopecia was found in almost half the population studied and it was more prevalent in those aged 20-39 years of age. The clinical pattern of hair loss was patchy followed by severe diffuse and mild diffuse. This pattern is in contrast to similar studies where the diffuse hair pattern of hair loss was more than the severe diffuse pattern [4,8,9]. We are unable to explain this difference in the pattern of hair loss as the patients in the Chanprapaph *et al*. study had a longer median SLE disease duration than our patients and their patients had a similar SLE hair loss duration to ours [9]. The authors opine that further studies to explore this relationship need to be conducted.

The common trichoscopy features observed in almost all the patients who had alopecia were; preserved brown honey comb pigment, hair thinning, decreased follicular units, hypopigmented hair, and short and lupus hair. Also observed were short re-growing hair and thick arborizing vessels in a few individuals, and follicular red dots in almost a quarter of the patients. The follicular red dots are reported to be telangiectatic vessels with extravasated erythrocytes reflecting on-going inflammation and vasculitis which suggests an active disease in these patients [9,10,19,20]. Trichoscopy of a normal pigmented scalp in richly pigmented scalp shows perifollicular brown honey comb pigment network and regular inter follicular white dots [20-22]. The honey comb pigment network is due to basal layer melanocytes and melanin, the white dots correspond to sweat duct and follicular openings [20-22]. A regular distribution of these white dots excludes scarring as was observed in these patients [22].

Follicular dots observed on trichoscopy were mostly brown scattered pigment, with a few black and blue/grey dots. Black dots signify broken hairs before emergence from the scalp or melanin dust while brown scattered pigments represent the presence of epidermal melanin [19,20]. Blue/grey dots represent pigment incontinence which can be confirmed on histopathology [19,20]. These trichoscopy features are in keeping with the non-scarring nature of the alopecia and in consonance with trichoscopy reports of non-scarring SLE hair loss from other studies [3,4,10,11].

On comparison with SLE patients without non-scarring alopecia, significant differences were observed in hair shaft and scalp pigmentary changes. The hair loss group had significant hair thinning, hypopigmented hairs, and short re-growing hair. They also, had a significant number with brown, blue/grey pigment, and black dots and only those with hair loss had follicular red dots. Although not attaining statistical significance, there was decreased follicular count in the hair loss group. These differences in hair shaft and follicular characteristics show that, despite SLE being a multi-system disease, the scalp may not be affected in patients who do not have hair loss. Ye *et al*.in their comparative study demonstrated similar to us follicular red dots in only alopecic SLE patients but Chanprapaph demonstrated it also in non-alopecic SLE patients [10,11].

Histopathology was conducted in a few patients. The commonest findings were epidermal atrophy, perivascular lymphocytic infiltrates, severe mucin deposition in over 40%, a reduction in size and number of sebaceous glands in almost 60%, increased telogen percentage, reduced anagen counts, reduced follicular counts, and preserved follicular units. Systemic LE typically targets anagen hair leading to a reduction in anagen hair and follicular hair count [9]. The decrease in sebaceous gland size and number in SLE patients is reported to be due to a chronic aggregation of inflammatory cells in the hair bulge area where the sebaceous glands are located [12,23]. Except for the reduction in the number and size of sebaceous glands, these histopathology findings are similar to what has been documented in the few histopathology studies of SLE non-scarring alopecia [9,11]. The reduction in sebaceous gland number found in this study is not usually reported in non-scarring alopecias [9,11]. We did not have a lot of interface changes contrary to what is commonly described [8,11]. The authors opine that, self-medication which is a common practice in the country may be responsible for this difference interface changes [24].

When histopathology features in those with and without alopecia were compared, the only significant difference was in the reduction in size and number of sebaceous glands. Mucin deposition was found irrespective of hair loss and there was no difference in the degree of mucin deposition in both groups. Mucin deposition is commonly reported in SLE and other connective tissue diseases and is said to represent an increase in ground substance [9,18]. Although we could not demonstrate a statistical difference in the severity of mucin deposition, severe mucin deposition was more in those who had alopecia. Though not attaining statistical significance, there was decreased follicular count in the hair loss group. This study shows that irrespective of hair loss, mucin deposition is common in SLE. It also confirms subtle differences between SLE patients with and without alopecia and confirms the decreased follicular counts observed clinically in alopecic patients.

We compared trichoscopy and histopathology features of the normal appearing scalp in the non-scarring hair loss group to the normal scalp of those without hair loss. On trichoscopy, the hair loss group had significantly more hypopigmented hair shafts and a decreased number of white dots. Although, not attaining to any significant difference, only the hair loss group had short re-growing hair. On histopathology, epidermal atrophy in half of the patients, perivascular lymphohistiocytic infiltrates, reduced size and a number of sebaceous glands, reduced follicular counts and increased telogen percentage were found in the normal appearing scalp of the hair loss group. This study demonstrates that in SLE patients, the normal appearing scalp is affected by the disease. The other comparative studies of SLE hair loss are silent on this [9-11].

We also compared the different types of non-scarring alopecia and found no significant differences on histopathology. The only significant trichoscopy difference was brown scattered pigment which was more in the severe diffuse hair loss. This study demonstrates that, histopathology and trichoscopy features of SLE non-scarring hair loss are independent of the degree or type of hair loss. Chanprapaph *et al*. following a similar study came to a similar conclusion [9].

In this study, there was a significant difference in SLE disease activity based on SLEDAI scores between those with and without hair loss. However, no significant difference was found in SLE disease activity between the different SLE hair loss types. Reports on SLE disease activity from studies are conflicted. In most studies, SLE disease activity is worse with hair loss [4,9-11] and in the study by Yun *et al*. there was no relationship between the two [8].

The strength of this study includes the comparative trichoscopy and histopathology evaluation between; patients with and without hair loss, normal appearing scalp of SLE non-scarring hair loss patients, and the scalp of patients without hair loss. This study was limited by being a one-center cross-sectional study, the number of patients available for study, and the number of patients that could be biopsied. The limited number of patients that could be biopsied makes the histopathology result not generalizable. Although SLE hair loss is well documented, this histopathology of it is not and this was one of the reasons for this study. We do not know if other researchers encountered hesitancy of scalp biopsy like our patients. Further studies on the histopathology of SLE hair loss are needed. In addition, it was difficult to completely rule out telogen effluvium due to stress from both the disease and its treatment.

## Conclusion

The prevalence of non-scarring alopecia is high in SLE and the commonest pattern is the patchy pattern. Systemic lupus erythematosus non-scarring hair loss is associated with trichoscopy (hair thinning, follicular red dots, pigmentary changes) and histopathology features (epidermal changes and reduction in size and number of sebaceous gland size). In addition, there are no differences in histopathology features between different types of SLE non-scarring hair loss but a difference in scalp pigmentation on trichoscopy. There are differences in trichoscopy and histopathology features between SLE patients with and without hair loss. Furthermore, the normal appearing scalp in SLE patients who have non-scarring hair loss is involved in the hair loss process. There is a significant difference in SLE disease activity between those with and without hair loss.

### What is known about this topic

- *Systemic lupus erythematosus is known to cause hair loss;*
- *Hair loss in SLE can be a sign of severity;*
- *One of the diagnostic criteria for SLE is hair loss*.

### What this study adds

- *There is a reduction in the number and size of sebaceous glands in SLE non-scarring alopecia;*
- *Microscopic involvement of the normal appearing scalp area in SLE patients predates their hair loss;*
- *There are differences in trichoscopy and histopathology features between SLE patients with and without hair loss*.

## Annexes

**Annex 1**: data collection proforma (PDF-114 Kb)

**Annex 2**: systemic lupus erythematosus disease activity index SLEDAI 2000 (PDF-109 Kb)
